# Modeling Temperature-Dependent Photoluminescence Dynamics of Colloidal CdS Quantum Dots Using Long Short-Term Memory (LSTM) Networks

**DOI:** 10.3390/ma17205056

**Published:** 2024-10-16

**Authors:** Ivan Malashin, Daniil Daibagya, Vadim Tynchenko, Vladimir Nelyub, Aleksei Borodulin, Andrei Gantimurov, Alexandr Selyukov, Sergey Ambrozevich, Mikhail Smirnov, Oleg Ovchinnikov

**Affiliations:** 1 Center for Continuing Education, Bauman Moscow State Technical University, 105005 Moscow, Russia; 2 P.N. Lebedev Physical Institute of the Russian Academy of Sciences, 119991 Moscow, Russia; 3 Scientific Department, Far Eastern Federal University, 690922 Vladivostok, Russia; 4 Department of Physics, Voronezh State University, 394018 Voronezh, Russia

**Keywords:** CdS, quantum dots, photoluminescence, temperature dependence, LSTM

## Abstract

This study addresses the challenge of modeling temperature-dependent photoluminescence (PL) in CdS colloidal quantum dots (QD), where PL properties fluctuate with temperature, complicating traditional modeling approaches. The objective is to develop a predictive model capable of accurately capturing these variations using Long Short-Term Memory (LSTM) networks, which are well suited for managing temporal dependencies in time-series data. The methodology involved training the LSTM model on experimental time-series data of PL intensity and temperature. Through numerical simulation, the model’s performance was assessed. Results demonstrated that the LSTM-based model effectively predicted PL trends under different temperature conditions. This approach could be applied in optoelectronics and quantum dot-based sensors for enhanced forecasting capabilities.

## 1. Introduction

Quantum dots (QDs) are semiconductor nanocrystals that have gained significant attention in recent years due to their unique optical and electronic properties, including strong photoluminescence (PL), making them highly suitable for applications in optoelectronics [1,2], along with organic and organometallic phosphors [3,4,5], bioimaging [6,7], and photovoltaic devices. The luminescence properties of QDs are known to be highly sensitive to temperature changes, influencing their emission intensity and wavelength.

### 1.1. Synthesis and Investigation of Properties of CdS QDs

Interest in CdS QDs began in the 1990s with the development of methods for their synthesis. Tsuzuki et al. [8] suggested in 1997 the first time a novel approach offered insights into CdS QD synthesis via mechanochemical reaction, with particle size controlled by grinding media size and extending their potential application. X-ray diffraction, transmission electron microscopy (TEM), and UV-VIS absorption spectroscopy confirmed the formation of ultrafine CdS QDs (<8 nm). Optical absorption exhibited a blue shift with decreasing particle size. The synthesis method involved a solid-state displacement reaction (${CdCl}_{2}+Na_{2}S\to CdS+2NaCl$) under a high-purity Ar gas atmosphere.

Chen et al. [9] were the first to synthesize water-soluble luminescent CdS quantum dots (QDs) capped with polyphosphate, L-cysteine, and thioglycerol in an aqueous solution. The polyphosphate-capped QDs demonstrated sensitivity to various cations, while the thioglycerol-capped QDs exhibited selective response to copper and iron ions. In contrast, the L-cysteine-capped QDs were specifically sensitive to zinc ions. These QDs offer selective ion detection capabilities, making them suitable for analyzing copper ions in the presence of physiological zinc concentrations.

Kim et al. [10] investigated the temperature dependence of PL dynamics in CdS QDs prepared by a colloidal method. Using size-selective photoetching and surface modification with a Cd(OH)_2_ layer, they obtained size-controlled CdS QDs with high PL efficiency. The PL decay profiles slowed down with increasing temperature, contrary to typical behavior. This anomalous temperature dependence is explained by a three-state model with a ground state, a lower-lying bound-exciton state, and a higher-lying free-exciton (dark-exciton) state with an optically inactive triplet nature.

Investigation of the effects of surface modification on the PL properties of CdS QDs prepared by a reverse-micelle method was explored by Kim et al. [11]. Modifying the QD surface with a Cd(OH)_2_ layer significantly enhanced the band-edge PL intensity of CdS QDs [12]. Initially, the PL-decay profile exhibited a fast decay component (<50 ps), which disappeared after modification. This drastic change in PL-decay profiles and increased PL intensity suggests that the enhancement originates from a significant reduction in nonradiative recombination processes due to the reduction in surface defects.

The preparation of luminescent CdS QDs modified with *l*-cysteine, demonstrating their water solubility and stability, was reported by Chen et al. [13]. The study systematically evaluates their potential as luminescence probes for heavy and transition metal ions in aqueous solutions. While silver ions enhance fluorescence emission without spectral shift, other bivalent ions like Hg^2+^, Cu^2+^, Co^2+^, and Ni^2+^ exhibit effective optical quenching effects on the QDs. Notably, CdS QDs show a red-shifted emission band when quenched by Hg^2+^ and Cu^2+^ ions. Under optimal conditions, the response to Ag^+^ ions follows a linear relationship with concentration, with a detection limit of $2.0\times 10^{-8}$ mol L^−1^. The concentration-dependent quenching effects of the other four ions are well described by the Stern–Volmer equation [14]. Overall, CdS QDs show promise as luminescence probes for detecting heavy and transition metal ions in aqueous solutions.

Zhen et al. [15] studied water-soluble mercaptoacetic acid-coated 3.1 nm CdS QDs to examine the correlation between PL and crystal growth mechanisms [16]. Using Ostwald ripening and oriented attachment (OA) growth mechanisms, they observed distinct differences in emission spectra evolution. Changes in surface and internal defects during OA crystal growth were responsible for the specific variations in PL of CdS QDs. Strategies for obtaining QDs with different luminescent properties are suggested [17].

CdS/ZnS QDs were synthesized using a water-in-oil microemulsion method by Fang et al. [18], with shell thickness controlled by the molar ratio of water to surfactant (R). The particle size increased with a higher R ratio [19]. Optical properties at room temperature were examined using absorption and PL spectra, revealing a strong blue shift in the PL peak as the R ratio [20] decreased. II-VI semiconductor QDs, including CdS, have garnered attention for their size-dependent optical properties, with core–shell structures like CdS/ZnS improving luminescence by surface passivation. CdS/ZnS QDs were prepared by forming a CdS core followed by adding Zn(NO_3_)_2_ to create the ZnS shell [21]. Absorption and luminescence spectra were measured at room temperature.

Field-induced changes in the optical absorption of CdS QDs were observed as the second-derivative of the absorption spectrum (Stark shift) and were described by Mehata [22] and indicated an enhanced electric dipole moment in the exciton state. The enhancement depended on the Q-dots’ shape and size. Field-induced PL changes revealed enhanced charge–transfer character and significant PL quenching due to exciton dissociation [23]. Understanding the QD character and field-induced PL quenching is important for applications in photovoltaics, LEDs, and biology.

The applications of QDs in optoelectronic devices often involve changes in the PL intensity and energy transfer processes that depend on excitation wavelength. Martynenko et al. [24] examined the excitation energy dependence (EED) of PL quantum yields [25] (QYs), decay kinetics, and circular dichroism [26] (CD) spectra of CdSe/CdS core/shell QDs with different shell thicknesses. The results revealed a strong correlation between the EED of PL QY and the zero-crossing points in CD profiles, suggesting exciton energy levels. This demonstrates the potential of CD spectroscopy to explore the electronic energy structure of chiroptically active nanocrystals with quantum confinement effects.

Smirnov [27] investigates non-radiative energy transfer from colloidal CdS QDs passivated with thioglycolic acid (CdS/TGA QDs) to methylene blue (MB) molecules across temperatures ranging from 300 to 80 K and reveals a decrease in CdS/TGA QDs luminescence lifetime with increasing MB^+^ concentration, indicating non-radiative resonance energy transfer [28]. Lowering the temperature to 80 K increases luminescence intensity and the lifetime of CdS/TGA QDs luminescence [29]. This suggests an effective Förster resonance energy transfer (FRET) system between CdS/TGA QDs and MB^+^ molecules.

Oxygen’s influence on the PL of CdSe/CdS core/shell QDs was systematically studied by Hu et al. [30]. With sufficient oxygen, QDs efficiently transitioned from inefficient trion to single-exciton states via deionization [31], facilitated by superoxide radicals. Increasing oxygen pressure accelerated deionization rates, stabilizing PL in both CdSe plain core and CdSe/CdS core/shell QDs. However, irreversible photocorrosion [32] occurred only in the CdSe plain core QDs, emphasizing the importance of high-quality epitaxial shells in QD applications.

Devadoss et al. [33] incorporated ${\mathrm{Ba}}^{2+}$ and ${\mathrm{Zn}}^{2+}$ ions into CdS. This enhances its optical properties without altering its structure. UV analysis reveals increased absorption intensity and a red-shifted band gap, attributed to quantum effects. PL emissions cover UV to red regions, with ${\mathrm{Ba}}^{2+}$ (2 wt. %) incorporation yielding vibrant colors. Characterization techniques confirm crystallinity and surface morphology, while elemental analysis verifies purity. ${\mathrm{Ba}}^{2+}$ and ${\mathrm{Zn}}^{2+}$ ion-incorporated CdS, synthesized simply without capping agents, holds promise for solar cells and LEDs, offering tunable emissions and UV absorption.

The ligand shell affects the stability and optical properties of CdS QDs in water colloidal solutions stabilized by 3-mercaptopropyltrimethoxysilane (MPS), and this was explored by Kuznetsova et al [34]. They established a correlation between stability and nanoparticle parameters like hydrodynamic radius [35], nucleus size [36], and zeta-potential [37] and identified optimal synthesis conditions [38] for stable colloidal solutions with luminescent properties. Additionally, they propose a mechanism for switching on the luminescence of MPS-capped CdS QDs in a water solution through pH control.

The exploration of CdS QDs has significantly advanced since their initial synthesis in the 1990s. A multitude of studies have been conducted to understand their synthesis, PL properties, and potential applications in various fields. These investigations range from novel synthesis techniques and surface modifications to the impacts of temperature and environmental factors on PL dynamics. Table 1 summarizes key studies that highlight the focus and results of these research efforts, showcasing the development and enhancement of CdS QDs for optoelectronic applications.

### 1.2. Challenges in Modeling Temperature-Dependent PL

PL behavior of CdS QDs during initial growth was studied by Kim et al. [11] alongside an annealing process. The heat-treated sample exhibited enhanced luminescence properties (quantum efficiency 29%) compared to the as-synthesized CdS QDs ( 1%), with a narrow band-edge emission. The annealing process effectively minimized accumulated defect states in the nanoparticles, leading to a reduction in nonradiative recombination [39]. This was corroborated by diffraction, absorption, and time-resolved PL measurements. This facilitated the production of highly luminescent and defect-free nanoparticles through a simple and straightforward process.

Fu et al. [40] examines carrier recombination mechanisms in perovskite solar cells to improve performance. A method combining steady-state and transient PL measurements quantifies key parameters such as trap density and carrier capture coefficients. Comparing methyl-ammonium lead iodide with a quadruple-cation composition, the findings suggest that the latter’s superior performance is linked to a reduced electron capture coefficient rather than lower trap density. The methodology involved simulating both steady-state and transient conditions using a general recombination model. Normalization of transient PL curves at 50 ns helped reduce uncertainties. Sensitivity analyses revealed significant variability in trap parameters across studies, indicating experimental uncertainties. The results show that steady-state PL intensity is sensitive to trap density, while transient PL curves vary based on parameters. Fitting a single recombination model to both data sets enhances accuracy in extracting trap properties, demonstrating the efficacy of this combined approach.

Yuan et al. [41] use PL measurements to analyze recombination dynamics in halide perovskites, which are vital for optimizing perovskite solar cells. Both steady-state and transient PL techniques are utilized to characterize the materials. Transient PL measurements are conducted using time-correlated single-photon counting and gated CCD systems. Excitation fluences are varied by employing filters with different optical density (OD) values, achieving a wide dynamic range. In addition, steady-state PL measurements are performed using a continuous wave laser (532 nm) to illuminate the samples, allowing for the recording of luminescence spectra to evaluate recombination dynamics and quasi-Fermi-level splitting. For numerical simulation, band diagrams for the devices are created using SCAPS software based on ultraviolet photoelectron spectroscopy (UPS) measurements. MATLAB scripts are developed to carry out PL simulations, incorporating coupled rate equations to model both steady-state and transient conditions effectively. This comprehensive approach enables a detailed analysis of the recombination processes occurring in the perovskite films, facilitating a deeper understanding of their performance in solar cell applications.

Zhang et al. [42] address the challenges in explaining the PL behavior of solution-processed methylammonium lead triiodide (MAPbI_3_) perovskite thin films, particularly concerning film thickness (L). PL decay kinetics exhibit bi-exponential behavior, where the fast ($\tau_{1}$) and slow ($\tau_{2}$) components relate to surface and bulk recombination, respectively. As L increases, both $\tau_{1}$ and $\tau_{2}$ show longer lifetimes, reflecting reduced trap density and improved charge transport. However, this complexity complicates the isolation of surface versus bulk contributions. Moreover, steady-state PL spectra show red shifts with increasing thickness, signaling changes in electronic environments and grain size that affect PL behavior. Thus, understanding these dynamics in MAPbI_3_ films requires a multifaceted approach combining PL measurements with other techniques.

Table 2 summarizes studies investigating the PL spectra of low-dimensional materials.

### 1.3. Aim of This Study

This study propose an approach by leveraging a machine learning (ML) technique, specifically forecasting PL spectra based on long short-term memory (LSTM) networks with hyperparameters optimized via Particle Swarm Optimiztion ((PSO) to develop predictive models based on existing PL spectra data. This study seeks to demonstrate the effectiveness of LSTM network in capturing this complex relationship and to provide a predictive model that can inform the design and optimization of QD-based devices. Ultimately, this research has the potential to advance the application across various QDs.

Research on LSTM-based forecasting of PL behavior in CdS QDs presents several notable contributions and novelties that can be generalized to other low-dimensional materials. First, LSTM models excel at handling time-series data, making them particularly suited for modeling dynamic behaviors like PL decay, quenching phenomena, and sensitivity to external influences such as temperature or pressure. This approach marks an improvement over traditional methods, such as exponential fitting, by learning temporal dependencies and non-linear relationships directly from the data without predefined assumptions.

A major challenge in CdS QDs, as in many low-dimensional materials, is the complexity of PL decay dynamics, often featuring multi-exponential or non-exponential characteristics due to surface states, quantum confinement effects, and trap-assisted recombination [43]. LSTM models are capable of capturing both fast and slow decay components, offering a more precise understanding of the underlying processes compared to conventional curve-fitting approaches [44]. This capacity to model complex decay patterns makes the LSTM approach valuable for other materials, such as perovskites, which also exhibit intricate PL behaviors.

Another contribution of LSTM-based forecasting is its ability to incorporate external experimental conditions—like excitation power, temperature, or environmental changes—into the model. This enables the accurate prediction of PL behavior under varying conditions, making the technique broadly applicable to optimizing material performance for a range of low-dimensional materials in devices such as LEDs, sensors, or solar cells.

Additionally, LSTM’s data-driven nature allows it to be trained on experimental data from different QD batches or synthesis methods, providing flexibility in learning from diverse datasets. This approach helps optimize material properties for improved PL performance, which is relevant for any material where experimental conditions or synthesis methods may vary. Consequently, LSTM-based models can significantly reduce experimental load by predicting PL outcomes, enabling researchers to explore material behavior without relying on time-consuming and costly experimental trials.

Finally, the generalizability of the LSTM method means it can be applied beyond CdS QDs to other QD systems, such as PbS or ZnSe, as well as to a wide variety of low-dimensional materials like 2D transition metal dichalcogenides (TMDs) or graphene QDs. The success of LSTM in forecasting PL behavior underscores its potential to become a universal tool for exploring optoelectronic properties across different material systems.

## 2. Materials and Methods

### 2.1. Experimental Setup

Water-soluble colloidal cadmium sulfide (CdS) quantum dots (QDs) were synthesized using a low-temperature aqueous method [45,46,47]. This involved injecting solutions of cadmium bromide (CdBr_2_) and sodium sulfide (Na_2_S) into a 3% inert photographic gelatin solution, which stabilized the nanoparticles. The growth of the QDs was controlled by halting the chemical reaction, allowing sizes to be tailored between 2.4 nm and 4.4 nm by varying the synthesis temperature from 40 °C to 90 °C.

Characterization techniques included UV–Vis absorption spectroscopy, revealing a broad absorption band from 2.68 to 3.45 eV, and PL measurements using an Ocean Optics Maya Pro 2000 system (Ocean Optics, Inc. World Headquarters 830 Douglas Ave., Dunedin, FL, USA 34698) with laser excitation at 380 nm and 405 nm. Transmission electron microscopy (TEM) confirmed the QD morphology, while X-ray powder diffraction (XRD) patterns obtained from an ARL X’TRA diffractometer (Thermo Fisher Scientific, Waltham, MA, USA) indicated a cubic zinc blende phase ($F\bar{4}3m$).

The morphology of the synthesized colloidal CdS QDs was characterized using a Carl Zeiss LEO 912 AB OMEGA (Carl Zeiss AG, Oberkochen, Germany) transmission electron microscope (TEM), enabling detailed structural analysis. The absorption spectrum of the colloidal CdS QDs was measured with a Perkin-Elmer Lambda 45 spectrophotometer (PerkinElmer, Inc., Shelton, CT, USA), providing insights into the optical properties of the QDs.

For the PL measurements, the CdS QD sample was placed in a cryostat where the pressure was reduced to $10^{-6}$ mbar using a Pfeiffer Vacuum HiCube 80 Eco (Pfeiffer Vacuum Technology AG, Asslar, Germany) turbomolecular pump. The PL spectra were recorded during heating at a constant rate of 0.05 K/s using an Ocean Optics Maya Pro 2000 spectrometer. A PicoQuant LDH-C 400 (PicoQuant GmbH, Berlin, Germany) pulsed laser, emitting at 405 nm, served as the excitation source for PL excitation.

### 2.2. Morphology and Optical Properties

**Figure 1:**
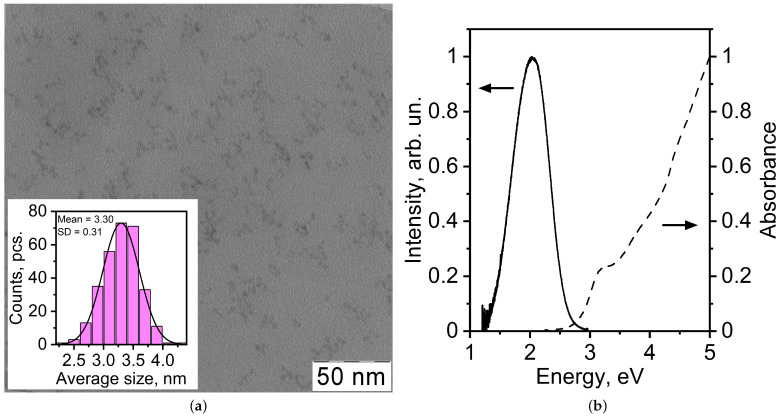
(**a**) TEM image of the synthesized semiconductor colloidal CdS QD. (**b**) Absorption (dashed curve) and luminescence (solid curve) spectra of CdS QD.

The synthesised QD were observed by transmission electron microscopy. Figure 1 shows the TEM-image of the synthesized CdS QDs. The synthesised nanoparticles had a spherical shape. Statistical analysis of the TEM-image showed that the CdS QDs have an average size 3.3 nm.

The absorption spectrum of CdS QDs (Figure 1) contains a local feature which is due to the interband transition. The local maximum of this feature is centered at the energy E = 3.19 eV. The maximum of the recombination PL of CdS QD (Figure 1) falls at an energy value of 2 eV with the FWHM of 0.7 eV. The maxima of PL and absorption spectra of colloidal CdS QD are shifted to the high-energy region relative to bulk CdTe crystal due to the quantum confinement effect [48,49].

The average size of the colloidal CdS QD was calculated from the first excitonic absorption peak using the following equation [50]:

(1) $$E_{QD}=E_{g}+\frac{\hbar^{2}\pi^{2}}{2\mu R^{2}}-\frac{1.78e^{2}}{\varepsilon R}-0.248E_{Ry}$$

where $E_{QD}$ and $E_{g}$ (2.36 eV) are bandgaps of the QDs and bulk crystal, respectively, ε = 5.5 [51], *R* is average size, $m_{e}$ = 0.205$m_{0}$ and $m_{h}$ = 1.6$m_{0}$ are the effective masses of electron and hole [52], $\mu =m_{e}m_{h}/\left(m_{e}+m_{h}\right)$, and $E_{Ry}=e^{4}/2\varepsilon^{2}\hbar^{2}\left(m_{e}^{-1}+m_{h}^{-1}\right)$ is is the effective Rydberg energy. The average size of the QDs was 3.2 nm.

### 2.3. Data Processing

**Figure 2:**
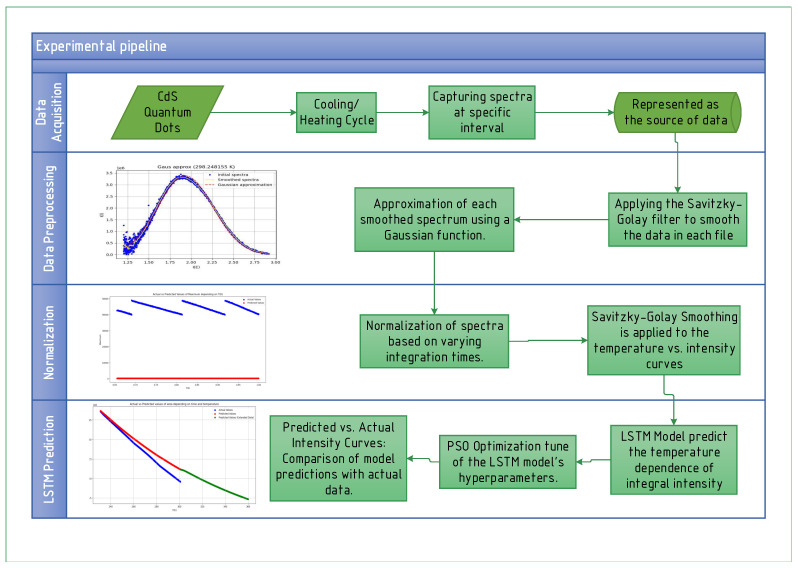
Workflow of the experimental and analytical approach used in this study.

In this study, spectra of CdS QDs were recorded using the experimental setup. On average, data acquisition occurred every 0.1±0.04 degrees, which corresponded to approximately 0.5±0.3 s per file. A total of two experiments were conducted. Initially, the QDs were rapidly cooled from room temperature to 86±1 K over a period of 15 min. Following this, the cooling process continued for approximately 20 min. The QD were then allowed to freely warm up to room temperature—240 K for the first experiment and 300 K for the second experiment. Figure 2 illustrates the workflow of our experimental and analytical approach.

In the first experiment, a total of 9770 files were recorded, while in the second experiment, 24,670 files were collected. The recorded spectra were analyzed as intensity versus energy, with the energy range spanning from 1.2 to 2.92 eV. Each spectrum was subsequently smoothed using the Savitzky–Golay filter with a window length of 50 and a polynomial order of 3. This choice of parameters was made to reduce noise while preserving the essential spectral features, ensuring accurate subsequent analysis.

**Figure 3:**
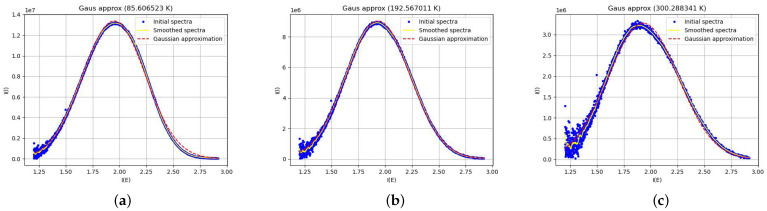
Examples of images of spectra with approximations at different temperatures. (**a**) 85.6 K, (**b**) 182.6 K, (**c**) 300.2 K.

Each smoothed spectrum was then approximated using a Gaussian function:

$$I\left(E\right)=A\exp\left(-\frac{{\left(E-B\right)}^{2}}{2C^{2}}\right),$$

where *A* represents the amplitude, *B* the peak energy, and *C* the width of the Gaussian. These coefficients were used to determine the integral intensity of the CdS QD as a function of temperature. Examples of images are shown in Figure 3.

However, given that this quantity is intensive and the integration time (in nanoseconds) of the setup varied during the experiments, all spectra were normalized by a scale factor to avoid abrupt changes in intensity. The resulting curves of integral intensity versus temperature were further smoothed using a Savitzky–Golay filter with a window length of 500 and a polynomial order of 1. Due to the large number of data points, a larger smoothing window was necessary.

Subsequently, these smoothed dependencies were utilized for LSTM-based predictions to model the temperature dependence of the integral intensity. To optimize the selection of LSTM hyperparameters, Particle Swarm Optimization (PSO) was employed. The PSO approach was chosen due to its effectiveness in exploring the hyperparameter space efficiently and its ability to balance exploration and exploitation, which impact achieving accurate predictions in this context. A detailed description of this methodology is provided in Section 2.4.

For LSTM training, the following features were selected: Time(s), representing the time elapsed since the beginning of heating when the QD were in a cooled state; T(K), denoting the temperature of the spectrum; and A, B, and C, which are coefficients of the approximating Gaussian. The target variable was defined as the ratio of the PL spectra integral intensity value to its maximum value, thereby normalizing the target variable.

All input features were also normalized using the MinMaxScaler from sklearn. This was performed to bring all features to the same scale, which is necessary for training a neural network. Training and testing datasets were created using a time window of size 500. This means that each record in the training dataset represents a sequence of 500 previous feature values, and the target variable represents the integral intensity value at the next step. The training data was split into training and testing sets in a ratio of 80% to 20%.

### 2.4. Optimization of LSTM Hyperparameters Using PSO

#### 2.4.1. LSTM Model

In this study, The PSO algorithm is applied to minimize the Mean Squared Error (MSE) of the LSTM model. The objective is to automate the process of tuning the hyperparameters of the model to improve its predictive capabilities. This approach is particularly relevant for time-series forecasting tasks, where the choice of optimal hyperparameters can significantly affect the model’s performance.

Given a dataset $D={\left\{\left(x_{t},y_{t}\right)\right\}}_{t=1}^{T}$, where $x_{t}$ is the feature vector at time *t*, and $y_{t}$ is the target variable, we aim to build a model ${\hat{y}}_{t}=f\left(x_{t};\theta \right)$ that minimizes the Mean Squared Error (MSE) between the predicted values ${\hat{y}}_{t}$ and the actual values $y_{t}$ of integral intensity.

The LSTM network is a type of recurrent neural network (RNN) designed to model sequential data. It addresses the vanishing gradient problem commonly encountered in traditional RNNs, enabling the network to learn long-term dependencies in the data. The core of the LSTM network is its memory cell, which can maintain its state over time, and the gating mechanisms that control the flow of information. Below, we detail the mathematical formulation of the LSTM unit.

An LSTM unit at time step *t* consists of the following components:

1. Memory Cell: The heart of an LSTM unit is the memory cell, which can store information over long periods. This allows the network to retain past information that might be crucial for making predictions about future data points.
2. Gating Mechanisms: LSTM networks utilize three types of gates to regulate the flow of information into and out of the memory cell:
  - Input Gate ($i_{t}$): This gate determines how much of the new information from the current input should be stored in the memory cell. It is controlled by a sigmoid activation function that produces values between 0 and 1, which helps to scale the input values.
  - Forget Gate ($f_{t}$): This gate decides what information from the memory cell should be discarded or retained. Similar to the input gate, it uses a sigmoid activation function to produce a value between 0 and 1 for each component of the memory cell, allowing the network to “forget” less relevant data.
  - Output Gate ($o_{t}$): This gate controls how much of the information in the memory cell is sent to the output of the LSTM unit, which important for making predictions based on the internal state of the network.

These gates are defined as follows:

$$i_{t}=\sigma \left(W_{i}\cdot \left[h_{t-1},x_{t}\right]+b_{i}\right),$$

$$f_{t}=\sigma \left(W_{f}\cdot \left[h_{t-1},x_{t}\right]+b_{f}\right),$$

$$o_{t}=\sigma \left(W_{o}\cdot \left[h_{t-1},x_{t}\right]+b_{o}\right),$$

where:

- $x_{t}$ is the input vector at time step *t*.
- $h_{t-1}$ is the hidden state from the previous time step t−1.
- $W_{i}$, $W_{f}$, and $W_{o}$ are weight matrices associated with the input, forget, and output gates, respectively.
- $b_{i}$, $b_{f}$, and $b_{o}$ are bias vectors for the input, forget, and output gates, respectively.
- σ(·) is the sigmoid activation function, which maps the input to a value between 0 and 1, thereby controlling the gate’s activation.

The cell state $C_{t}$ is updated by combining the previous cell state $C_{t-1}$, modulated by the forget gate, with the new candidate cell state ${\tilde{C}}_{t}$, modulated by the input gate:

$$C_{t}=f_{t}⊙C_{t-1}+i_{t}⊙{\tilde{C}}_{t},$$

where:

- ⊙ denotes element-wise (Hadamard) multiplication.
- ${\tilde{C}}_{t}$ is the candidate cell state, which is computed as:
  $${\tilde{C}}_{t}=\tanh\left(W_{C}\cdot \left[h_{t-1},x_{t}\right]+b_{C}\right),$$
  where $W_{C}$ is the weight matrix and $b_{C}$ is the bias vector associated with the candidate cell state, and tanh(·) is the hyperbolic tangent function, which maps the input to a value between −1 and 1.

The forget gate $f_{t}$ decides how much of the previous cell state $C_{t-1}$ should be retained, while the input gate $i_{t}$ determines how much of the new candidate state ${\tilde{C}}_{t}$ should be added to the cell state.

The hidden state $h_{t}$ is computed as:

$$h_{t}=o_{t}⊙\tanh\left(C_{t}\right),$$

where the output gate $o_{t}$ modulates the output of the cell state $C_{t}$. This hidden state $h_{t}$ is used both as the output of the LSTM cell at time step *t* and as input to the next time step t+1.

The LSTM network thus achieves its ability to learn long-term dependencies through the regulation of information flow via its gating mechanisms. By combining past information (through the forget gate) with new inputs (through the input gate) and controlling the output (through the output gate), the LSTM network is capable of modeling complex temporal patterns in sequential data.

#### 2.4.2. PSO-Based Hyperparameters Tuning

PSO is a population-based optimization algorithm inspired by the social behavior of birds flocking or fish schooling. The algorithm is used to optimize a problem by iteratively improving a candidate solution with regard to a given measure of quality, which in this case is the MSE of an LSTM model.

PSO algorithm initializes a swarm of particles, where each particle represents a potential solution in the hyperparameter space θ. Let the position of the *i*-th particle in the *d*-dimensional hyperparameter space at iteration *t* be denoted by $p_{i}^{t}=\left[\theta_{i,1}^{t},\theta_{i,2}^{t},\ldots ,\theta_{i,d}^{t}\right]$. The velocity of the *i*-th particle is represented by $v_{i}^{t}=\left[v_{i,1}^{t},v_{i,2}^{t},\ldots ,v_{i,d}^{t}\right]$.

At each iteration, the velocity and position of each particle are updated based on its own experience and the experience of its neighbors, according to the following equations:

$$v_{i}^{t+1}=\omega v_{i}^{t}+c_{1}r_{1}\left(p_{i}^{\mathrm{best}}-p_{i}^{t}\right)+c_{2}r_{2}\left(g^{\mathrm{best}}-p_{i}^{t}\right),$$

$$p_{i}^{t+1}=p_{i}^{t}+v_{i}^{t+1},$$

where:

- ω is the inertia weight, controlling the influence of the previous velocity on the current velocity. It helps balance exploration (searching new areas) and exploitation (refining known good areas).
- $c_{1}$ is the cognitive coefficient, representing the particle’s tendency to return to its own best position $p_{i}^{\mathrm{best}}$ found so far.
- $c_{2}$ is the social coefficient, representing the particle’s tendency to move toward the global best position $g^{\mathrm{best}}$ found by the entire swarm.
- $r_{1}$ and $r_{2}$ are random numbers uniformly distributed in the interval [0,1], which introduce stochastic behavior to the particle’s movement.

The particles iteratively adjust their positions in the search space according to the above rules, moving towards regions of the search space that are likely to yield better solutions. The quality of each particle’s position is evaluated using the objective function, which in this case is the Mean Squared Error (MSE) calculated on the validation set of the LSTM model:

$$\mathrm{MSE}\left(\theta \right)=\frac{1}{N}\sum_{i=1}^{N}{\left(y_{i}-{\hat{y}}_{i}\right)}^{2},$$

where ${\hat{y}}_{i}$ are the predictions of the LSTM model, and $y_{i}$ are the true values.

For this study, the PSO algorithm was applied to optimize three key hyperparameters of the LSTM model:

1. The number of LSTM units per layer units.
2. The number of training epochs epochs.
3. The batch size batchsize used during training.

The search space for these hyperparameters was defined with the following bounds:

units∈[10,100],epochs∈[1,10],batchsize∈[16,128].

These ranges were chosen based on prior knowledge and experimental considerations.

PSO algorithm was implemented with a swarm size of *S* particles, and the optimization process was run for *I* iterations. The choice of *S* and *I* affects the convergence speed and the likelihood of finding the global minimum. Typical values for *S* and *I* are determined experimentally, balancing computational cost and solution quality.

At each iteration:

1. Each particle’s position (set of hyperparameters θ) is evaluated using the MSE objective function.
2. The personal best position $p_{i}^{\mathrm{best}}$ and global best position $g^{\mathrm{best}}$ are updated if the current position yields a lower MSE.
3. The particles’ velocities and positions are updated based on the equations provided above.

The algorithm terminates after *I* iterations, or if the improvement in the global best MSE falls below a pre-defined threshold.

Upon completion, the PSO algorithm provides the best set of hyperparameters $\theta^{*}=\left\{{\mathrm{units}}^{*},{\mathrm{epochs}}^{*},\mathrm{batch}\;{\mathrm{size}}^{*}\right\}$ that minimize the MSE. These optimal parameters are then used to train the final LSTM model, which is expected to provide the most accurate predictions on the test dataset.

Thus, the proposed approach effectively automates the process of selecting hyperparameters for the LSTM model, leading to improved time-series modeling and reduced Mean Squared Error.

## 3. Results

### 3.1. Temperature Dependencies

**Figure 4:**
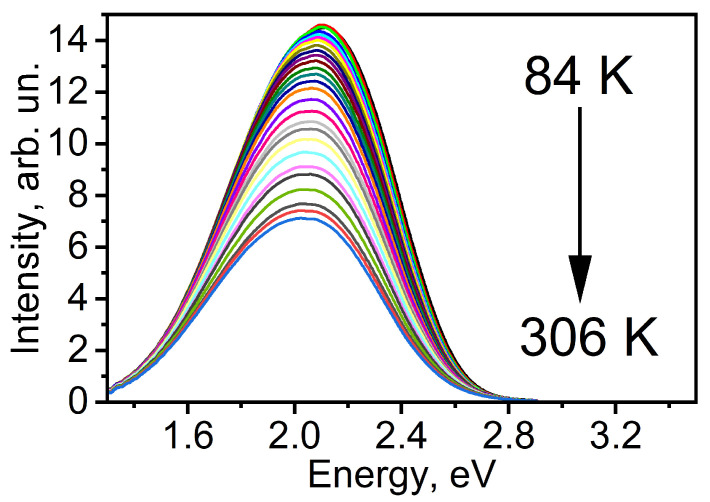
The PL spectra of CdS quantum dots (QDs) were measured as the sample was heated from 84 K to 306 K, with curves recorded at intervals of 7 K.

Figure 4 shows the PL spectra of colloidal CdS QD at different temperatures. It can be seen that with increasing temperature there is a quenching of the PL intensity and a redshift of the PL spectrum.

**Figure 5:**
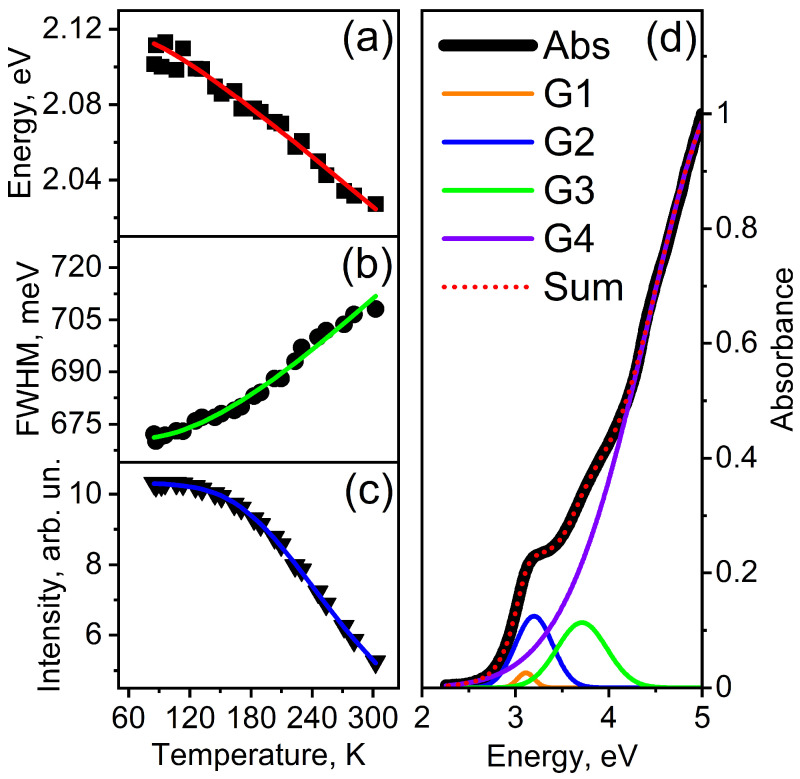
Temperature-dependence of trap state luminescence peak energy (**a**), FWHM (**b**), and integrated intensity (**c**) for CdS QDs. The approximation of the absorption spectrum by four Gaussian functions (G1–G4); the red dashed line shows the fitting result (**d**).

Nowadays, many types of theoretical and empirical equations are known to fit the temperature dependence of the interband luminescence peak of different nanostructures. Some authors employed the Varshni’s equation to fit the temperature dependence of the trap state luminescence peak [53,54]. In a number of cases, the fitting parameters in the Varshni’s equation are negative [55], which generally complicates the physical interpretation of the recorded dependences. In our study, we used the Fan’s equation [56,57,58] to fit the temperature dependence of the trap state luminescence peak (Figure 5a), since the Varshni’s equation gave a large fitting error.

(2) $$E_{g}\left(T\right)=E_{g}\left(0\right)-\frac{A_{F}}{e^{\theta /T}-1}$$

where $E_{g}\left(T\right)$ is the bandgap energy of QDs at a specific temperature *T*, $E_{g}\left(0\right)$ is the bandgap energy of QDs at 0 K, $A_{F}$ is the Fan’s parameter that depends on the microscopic characteristics of materials [56,57], $\theta =\hbar \omega /k_{B}$ is an average phonon temperature, and $k_{B}$ is the Boltzmann constant. The red line in Figure 5a shows the simulation of bandgap energy exploiting Equation (2). We obtain the best-fit curve for $A_{F}$ = 112 meV and θ = 235 K. The temperature coefficient of the energy-level shift expressed as $\beta =A_{F}/\theta$ under the following condition $k_{B}T\gg \hbar \omega$ [56]. The calculation showed that $\beta =4.7\cdot 10^{-4}$ eV/K. These values are consistent with the parameters for bulk CdS: $\beta =\left(3.9-4.4\right)\cdot 10^{-4}$ eV/K [10,59] and θ=(219−300) K [10,53,60]. The calculated result indicates that the observed temperature dependence is an intrinsic optical characteristic of the CdS material.

The full width at half maximum (FWHM) of the trap state luminescence (Figure 5b) continuously increases with the sample temperature. To gain a deeper understanding of the carrier–phonon scattering processes contributing to the observed broadening, fitted the experimental data to a relation describing the temperature dependence of the broadening of luminescent peaks of nanoparticles [53]

(3) $$\Gamma \left(T\right)=\Gamma_{0}+\sigma T+\frac{\Gamma_{LO}}{e^{E_{LO}/k_{B}T}-1}$$

where $\Gamma_{0}$ represents a temperature-independent non-uniform broadening, arising from variations in the composition, shape, and size of the nanoparticles; σ is the coefficient of the interaction of excitons with acoustic phonons; and $\Gamma_{LO}$ reflects the strength of interaction of excitons with longitudinal optical phonons with energy $E_{LO}$. The LO phonon energy $E_{LO}$ = 38 meV is borrowed from the study of Ye et al. and kept fixed for the fitting [61]. The green line in Figure 5b shows the simulation of FWHM exploiting Equation (3). The optimal curve corresponds fairly closely to the experimental data for $\Gamma_{0}=670$ meV, σ=1.3 μeV, and $\Gamma_{LO}=135$ meV. These values indicate that the contribution of LO phonons is much larger than that of acoustic phonons, which is consistent with the calculations of Rudin et al. [62,63].

Figure 5c shows the dependence of the integrated PL intensity of CdS QD on temperature. Taking into account two primary non-radiative relaxation processes, namely carrier capture by surface defect states [64] and multiple longitudinal optical phonon-assisted thermal escape [65], the temperature dependence of the PL intensity can be modeled using the following equation [66].

(4) $$I\left(T\right)=\frac{I_{0}}{1+Ae^{-E_{a}/k_{B}T}+B{\left(e^{E_{LO}/k_{B}T}-1\right)}^{-m}}$$

The parameter $I_{0}$ denotes the initial PL intensity at 0 K. The symbol $E_{a}$ signifies the activation energy associated with thermal quenching. The variable *m* indicates the number of LO phonons involved in the thermal escape of carriers. The constants *A* and *B* are the ratios of the radiative lifetime to the capture time of non-radiative recombination centers, respectively. The blue line in Figure 5c shows the simulation of integrated intensity of trap state luminescence exploiting Equation (4). We obtain the best-fit curve for $E_{a}$ = 21.2 meV and *m* = 2.4. The LO phonon energy was fixed at $E_{LO}$ = 38 meV. The activation energy value calculated by us agrees with the activation energy for trap state luminescence of CdS QD, which was obtained in a study [67]. The decrease in the integrated intensity of trap state luminescence of CdS QDs can be explained by the thermal escape assisted by LO phonon scattering.

The origin of the value of *m* can be understood by estimating the energy values of the first four excited states from the absorption spectrum of colloidal semiconductor CdS QD. The absorption spectrum and fit by four Gaussian functions are shown in Figure 5d. The energy difference between the first two resonance absorption states is around 87.1 meV, which is close to the 2$E_{LO}$ energy. Hence, it can be concluded that the thermal escape is due to the scattering of two LO phonons [65].

The absorption spectrum shown in Figure 5d is modeled using four Gaussian functions labeled G1, G2, G3, and G4. The black line represents the experimentally measured absorption, plotted as absorbance against energy in electron volts (eV). To approximate the measured spectrum, four Gaussian functions are used to capture different features or peaks in the spectrum.

Each Gaussian function corresponds to a distinct absorption peak or contribution within the spectrum. For instance, G1 (orange) represents a small peak around 3 eV, while G2 (blue) captures a broader peak near 4 eV. G3 (green) is a sharper peak around 3.5 eV, and G4 (purple) accounts for the large, broad peak at higher energies, extending towards 5 eV.

These Gaussian functions are summed together to produce the red dotted line, which represents the combined fit to the experimental data. The fitting process involves adjusting the position, width, and height of each Gaussian function to closely match the overall absorption profile. By fitting the spectrum with this sum of Gaussians, different physical processes or states contributing to the absorption can be separated and analyzed individually.

In essence, this approach of using multiple Gaussian functions allows the complex absorption spectrum to be broken down into simpler, identifiable components, making it easier to understand the different underlying optical processes.

### 3.2. LSTM Results

**Figure 6:**
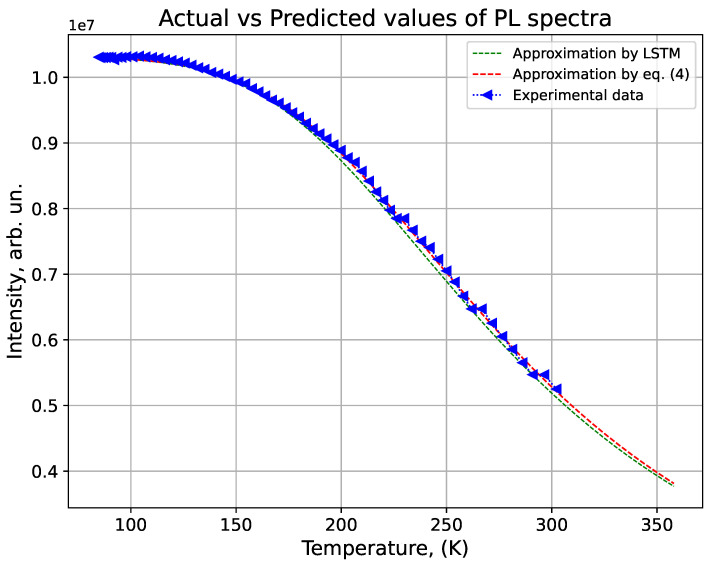
LSTM prediction results.

Figure 6 shows the dynamics of PL in CdS QDs as a function of temperature, with three different curves: experimental data, an approximation using Equation (4), and a prediction based on an LSTM model. As the temperature increases from 100 K to 350 K, the intensity of PL is tracked to compare how well each model aligns with the actual measurements.

The experimental data provide the real behavior of the CdS QDs’ PL intensity, serving as a reference point for the theoretical and predictive models. The approximation based on Equation (4) offers a theoretical prediction derived from a physical model that depends on the parameter $E_{LO}$, typically determined through additional experiments like Raman spectroscopy.

In contrast, the LSTM model takes a data-driven approach, predicting PL behavior based solely on historical data trends, without requiring extra experiments. A key advantage of the LSTM model is its ability to make accurate predictions without needing to determine the $E_{LO}$ coefficient from Raman spectroscopy, which is necessary for the theoretical model.

This makes the LSTM model a more efficient and cost-effective tool, as it can predict the photoluminescence dynamics of CdS QDs without the need for labor-intensive physical experiments, offering a streamlined alternative to traditional methods.

The LSTM model shows a strong correlation between actual and predicted values in the temperature range from 84 K to 180 K, demonstrating high prediction accuracy within this interval. However, there is a slight deviation from the experimental data and the theoretical model between 180 K and 300 K, indicating a minor reduction in predictive precision at higher temperatures. In the extended range (300–360 K), a decrease in integrated intensity is observed, which the model successfully forecasts, although no experimental data is available in this region.

The coefficient of determination $R^{2}$ between the theoretical model and the LSTM predictions is 0.98, indicating a high level of agreement between these approaches and confirming the effectiveness of the LSTM model in predicting the PL of CdS quantum dots.

These results were obtained using an LSTM model with parameters epochs = 4, batch size = 32, and units = 50, which were optimized using PSO to minimize MSE. The optimization allowed the selection of parameters that ensured the best match between actual and predicted values. The low MSE value confirms the high accuracy of the LSTM model for predicting the integrated PL intensity.

The relatively small number of epochs, 4, indicates that the model converges quickly and achieves good results without overfitting. The batch size of 32 allowed efficient weight updates during training, while 50 hidden units in the LSTM layer provided the necessary flexibility to model the complex temporal dependencies in the data.

The use of the PSO for hyperparameter optimization proved to be a successful approach. The PSO helped find a set of parameters that minimized MSE, which in turn led to higher prediction accuracy. This optimization technique has shown its usefulness in time-series prediction tasks, such as the analysis of PL dynamics.

A noteworthy aspect is that the LSTM model, trained on data up to 300 K, was able to reasonably predict the system’s behavior in the extended temperature range from 300 to 360 K (green points on the graph). This demonstrates that the model not only learned well from the available data but also generalized effectively. However, it should be noted that there are no experimental data in this range for comparison, so these predictions require further validation.

## 4. Discussion

### 4.1. Advantages and Limitations of Proposed Approach

This study presents an approach to modeling the temperature-dependent PL of CdS colloidal QD using LSTM networks. The findings highlight the potential of LSTM networks in accurately predicting PL behavior across various temperature conditions, offering significant implications for optoelectronics and quantum dot-based sensors.

One of the key strengths of the LSTM-based model is its ability to handle temporal dependencies effectively [68]. Traditional modeling approaches often struggle with capturing the dynamic nature of PL properties that vary with temperature. LSTM networks, on the other hand, are specifically designed to manage sequential data and can capture complex temporal patterns, making them well-suited for this application.

The integration of time-series data of PL intensity with corresponding temperature variations allows the LSTM model to learn and predict future trends accurately. This capability is essential for applications where real-time monitoring and prediction of PL behavior are essential, such as in optoelectronic devices and sensors.

However, the efficacy of LSTM models is contingent upon the quality and quantity of training data. In practice, gathering extensive, high-quality datasets can be challenging, especially for novel materials or under varying experimental conditions [69]. If the dataset is insufficient or biased, the model’s predictive performance could suffer, leading to inaccuracies in understanding PL behavior. Future work must address these limitations by employing robust data collection strategies and possibly augmenting datasets to ensure comprehensive training for LSTM models.

Despite the promising findings, several limitations and challenges remain. A significant concern is the interpretability of LSTM models [70]. While these models excel in making predictions, their “black box” nature can obscure the underlying mechanisms driving PL behavior, complicating the extraction of meaningful insights regarding the material’s physics [71]. This lack of interpretability poses challenges in identifying fundamental processes essential for guiding further experimental work. This makes it difficult to extract clear, interpretable insights from the LSTM’s inner workings or to understand why the model behaves differently under certain conditions, such as the slight deviation in predictive accuracy at higher temperatures. Although this study serves as a preliminary exploration of LSTM’s application in this domain, its broader scientific impact may be limited. Nonetheless, it provides value in specific scenarios where modeling without deep physics insights can be advantageous.

While the model offers practical advantages, such as the ability to bypass additional experiments like Raman spectroscopy, this lack of transparency poses a limitation. It is harder to validate the model’s internal logic or generalize its predictions beyond the data it was trained on, unlike theoretical models that can be adjusted or re-derived based on physical laws. To address these interpretability issues, further research into methods like attention mechanisms, explainable AI (XAI) approaches, or hybrid models that combine physics-based and data-driven methods could help bridge the gap, offering both high prediction accuracy and clearer insight into the model’s decision-making process. One of the relevant XAI methods is SHAP (SHapley Additive exPlanations) [72,73]. Its values provide a consistent way to interpret the contribution of each feature (such as temperature or past luminescence values) to the model’s predictions. By calculating the marginal contribution of each feature to the final output, SHAP values can help explain which variables have the greatest influence on the LSTM’s predictions across different temperature ranges.

To address these interpretability issues, further research into methods like attention mechanisms, explainable AI (XAI) approaches, or hybrid models that combine physics-based and data-driven methods could help bridge the gap, offering both high prediction accuracy and clearer insight into the model’s decision-making process.

Additionally, the reliance of LSTM models on hyperparameter tuning presents a significant challenge. Achieving optimal performance necessitates the careful selection of various parameters, including learning rates, batch sizes, and network architectures. This process can be time-consuming and often demands extensive experimentation. To address this issue, this study has employed PSO for hyperparameter optimization (HPO), aiming to streamline the tuning process and enhance the overall efficiency of the model.

Finally, while the findings demonstrate the versatility of LSTM in modeling PL behavior, validation against experimental data is essential. Continuous refinement of the model based on experimental feedback will enhance its accuracy and reliability. This iterative process will contribute to the advancement of LSTM methodologies in the field.

The accurate prediction of PL behavior is particularly valuable in the field of optoelectronics [74]. Optoelectronic devices, such as light-emitting diodes (LEDs) and photovoltaic cells, often rely on the stable and predictable performance of QDs. By using the LSTM-based model, engineers can better design and optimize these devices to operate efficiently under varying temperature conditions. This can lead to improved performance, reliability, and longevity of optoelectronic components.

Quantum dot-based sensors are used in various applications, including environmental monitoring [75], biomedical diagnostics [76], and industrial process control [77]. The ability to predict PL behavior accurately can enhance the sensitivity and selectivity of these sensors. For example, in environmental monitoring, predicting the response of QD to temperature changes can improve the accuracy of gas and pollutant detection.

Moreover, the LSTM model can be extended to other types of low-dimensional materials and different environmental factors, such as humidity or pressure, further expanding its applicability in sensor technology.

**Figure 7:**
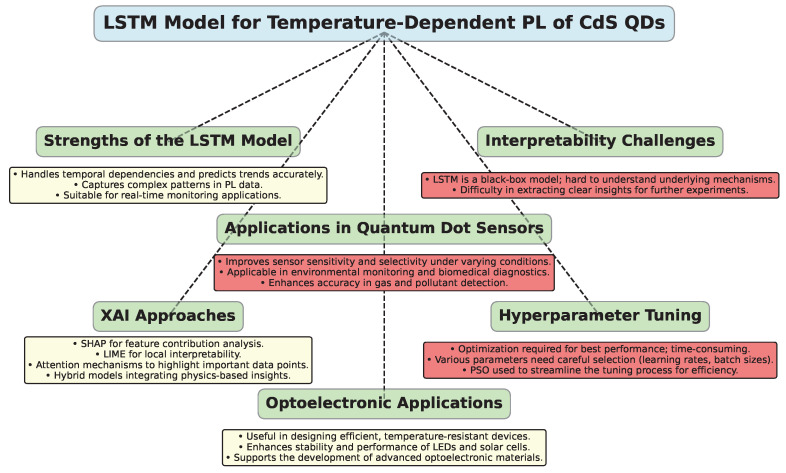
Overview of the LSTM model for predicting temperature-dependent PL of CdS QDs.

The diagram in Figure 7 highlights the key strengths, challenges, and applications of the LSTM model for predicting temperature-dependent PL of CdS QDs.

### 4.2. Future Directions

While the LSTM-based model shows promising results, there are several avenues for future research that could enhance its predictive capabilities and extend its application to a broader range of conditions.

One potential direction is the inclusion of more environmental variables that affect the PL properties of CdS QDs. Currently, the model uses temperature as a key variable, but PL can also be influenced by other factors such as light intensity because variations in the excitation source’s intensity could affect the quantum dot’s PL yield [78]. Modeling this could allow for better predictions under varying experimental conditions. QDs are often sensitive to the surrounding chemical environment, including the pH level [79]. Including pH data in the model might improve its accuracy in biological or chemical sensing applications. Some QDs exhibit changes in their PL when interacting with particular chemicals or gases [80]. Incorporating these interactions into the model could enable predictive capabilities in areas like environmental monitoring or chemical detection. By collecting and integrating data on these additional factors, the LSTM model could become a more comprehensive predictive tool, applicable to a wider range of experimental or practical settings. This multi-dimensional data approach would also allow researchers to explore more complex relationships between environmental conditions and the PL response of QDs.

Another key area of future research is the further optimization of the LSTM network architecture. In this study, the LSTM model employed a specific configuration of epochs, batch size, and units, but different configurations could yield even better results. Several architectural aspects could be explored, such as number of LSTM layers. A deeper network with more layers could potentially capture more complex patterns in the data [81]. However, this comes with the risk of overfitting or increased computational cost, so careful tuning would be necessary. Increasing or decreasing the number of neurons in each LSTM layer could affect the model’s ability to generalize [82]. Finding the optimal number of neurons might further improve performance, especially for datasets with more variability or noise. The learning rate, batch size, and number of epochs can all have significant impacts on the model’s performance. Techniques such as grid search, random search, or more advanced methods like Bayesian optimization could be employed to systematically explore and fine-tune these hyperparameters [83].

Furthermore, advanced training techniques like dropout [84] or batch normalization [85] could be introduced to prevent overfitting and improve the model’s ability to generalize across unseen data. This would be especially useful when dealing with more extensive datasets or extended prediction ranges, as seen in this study’s temperature range extension.

To further enhance prediction capabilities, the LSTM model could be combined with other machine learning techniques, such as Convolutional Neural Networks (CNNs). CNNs excel at extracting spatial features and could be used in conjunction with LSTM to analyze spectral data or other two-dimensional inputs like PL intensity maps [86,87]. This hybrid approach, known as ConvLSTM [88,89], has shown success in other domains like video analysis and weather forecasting and could be adapted for PL prediction. Reinforcement Learning (RL) could be used to continuously optimize the LSTM model as new data become available [90]. For instance, the model could be trained to predict optimal environmental conditions (temperature, pH, etc.) for maximizing PL, with rewards based on the accuracy of its predictions or the efficiency of its operation. Combining the LSTM model with other machine learning models like Random Forests [91], Gradient Boosting [92], or even different neural network [93] architectures could create a more versatile and robust predictive tool. Ensemble learning could help by leveraging the strengths of various models, leading to improved accuracy and reduced prediction error.

In cases where experimental data are limited, transfer learning could be applied [94]. The LSTM model trained on a dataset of CdS QD could be fine-tuned to predict the behavior of different QD materials, such as CdSe or ZnS, with relatively few additional training data points. Domain adaptation could also be explored to apply the model trained in one experimental condition to another, such as transitioning between different light sources or QD synthesis techniques.

By exploring these avenues—integrating additional variables, optimizing the LSTM architecture, combining with other machine learning techniques, and leveraging transfer learning—the predictive capabilities of the LSTM model for PL behavior could be significantly enhanced. This would enable more accurate and comprehensive predictions, not only for CdS QD but potentially for other nanomaterials and applications across a variety of fields. These enhancements would open the door for real-time, adaptive predictive models that could be applied in environments like optoelectronics, bioimaging, and environmental sensing.

## 5. Conclusions

This study demonstrates the effectiveness of LSTM networks in modeling the temperature-dependent PL of CdS colloidal QDs. The LSTM-based model offers a powerful tool for predicting PL trends, with implications for optoelectronics and quantum dot-based sensors. Future research should focus on expanding the model’s capabilities and exploring its applications in other areas of QD technology. The integration of advanced ML techniques with QD research holds great promise for developing innovative and efficient technologies.

Additionally, the findings indicate that the PL decay behavior of CdS QDs is significantly influenced by surface traps and defect states, which contribute to the complexity observed in PL dynamics. The study highlights that as the excitation fluence varies, the interplay between surface and bulk recombination processes can be effectively modeled using LSTM, leading to improved insight into how these factors impact overall PL efficiency.

The correlation between model predictions and experimental data emphasizes the importance of considering more types of variables when analyzing PL dynamics. By establishing an understanding of this relationship, the study sets a solid foundation for optimizing PL behavior in CdS QDs and other similar materials.

The primary novelty of this study lies in the application of LSTM networks for modeling PL behavior, a methodology not previously utilized in the context of low-dimensional materials. This approach offers a transformative perspective on data analysis in materials science, enabling the extraction of meaningful insights from complex time-series data. The successful demonstration of LSTM’s capabilities not only contributes to the field of optoelectronics but also provides a roadmap for future research involving other quantum materials and nanostructures.

Based on the obtained results, several recommendations can be made for future research directions:

- Researchers may explore the application of LSTM models to other low-dimensional materials, such as perovskites, transition metal dichalcogenides (TMDs), and organic semiconductors. This expansion will validate the generalizability of LSTM techniques across diverse material systems and enable comparative studies to enhance the understanding of PL dynamics.
- Future studies could incorporate additional experimental variables, such as temperature, excitation wavelength, and environmental conditions, to further refine LSTM predictions. The inclusion of these factors can provide a more comprehensive view of the conditions affecting PL behavior and optimize device performance.
- The integration of LSTM with other analytical techniques, such as machine learning methods and experimental diagnostics (e.g., time-resolved spectroscopy), may yield a more holistic approach to understanding PL dynamics. This combination can facilitate the development of predictive models that not only forecast PL behavior but also provide insights into underlying physical mechanisms.
- Given the identified influence of trap states on PL behavior, future research should aim to investigate methods for reducing defect densities through advanced synthesis techniques or post-synthesis treatments. By mitigating trap states, researchers can enhance the efficiency of optoelectronic devices.
- Developing real-time monitoring systems based on LSTM predictions can assist in the optimization of QD synthesis and processing conditions, allowing for in situ adjustments to improve PL performance.

## Figures and Tables

**Table 1:** Summary of studies on CdS QDs.

Reference	Focus	Results
Tsuzuki et al. [8]	Synthesis of CdS QDs via mechanochemical reaction	Developed a novel approach to synthesize ultrafine CdS QDs (<8 nm) with controlled particle size; confirmed by XRD, TEM, and UV-VIS absorption spectroscopy.
Chen et al. [9]	Water-soluble luminescent CdS QDs	First synthesized water-soluble CdS QDs with selective ion detection capabilities; sensitivities varied with capping agents (e.g., polyphosphate, thioglycerol).
Kim et al. [10]	Temperature dependence of PL dynamics	Anomalous PL decay profiles slowed with increasing temperature, explained by a three-state model; demonstrated size-controlled QDs with high PL efficiency.
Kim et al. [11]	Effects of surface modification on PL properties	Enhanced band-edge PL intensity through Cd(OH)_2_ layer modification; significant reduction in nonradiative recombination processes noted.
Chen et al. [13]	Luminescent CdS QDs modified with L-cysteine	Demonstrated potential as luminescence probes for metal ions in solutions; detection limits and concentration-dependent quenching effects were characterized.
Zhen et al. [15]	Correlation between PL and crystal growth mechanisms	Identified emission spectrum evolution related to Ostwald ripening and oriented attachment; variations in PL attributed to surface and internal defects.
Fang et al. [18]	Synthesis of CdS/ZnS QDs using microemulsion method	Demonstrated control over shell thickness and observed strong blue shift in PL peaks as the water-to-surfactant ratio changed.
Mehata [22]	Field-induced changes in optical absorption of CdS QDs	Observed enhanced electric dipole moments and significant PL quenching due to exciton dissociation; insights for applications in various technologies.
Martynenko et al. [24]	Excitation energy dependence of PL quantum yields	Established strong correlation between excitation energy and PL quantum yields; potential for using circular dichroism to study electronic structures in QDs.
Smirnov [27]	Non-radiative energy transfer from CdS QDs to MB molecules	Revealed effective Förster resonance energy transfer; luminescence intensity and lifetime improved at lower temperatures.
Hu et al. [30]	Influence of oxygen on the PL of CdSe/CdS QDs	Showed efficient transition from trion to single-exciton states with increasing oxygen pressure; highlighted the significance of high-quality epitaxial shells.
Devadoss et al. [33]	Optical properties of CdS with Ba^2+^ and Zn^2+^ incorporation	Enhanced optical properties and tunable emissions were achieved; confirmed structural integrity and purity without capping agents.
Kuznetsova et al. [34]	Stability and optical properties of CdS QDs in water solutions	Correlated stability to hydrodynamic radius and zeta-potential; proposed a pH control mechanism to switch on luminescence in MPS-capped CdS QDs.

**Table 2:** Summary of PL studies in materials.

Reference	Focus	Material	Applied Method	Limitations	Results
Fu et al. [40]	Carrier recombination mechanisms in perovskite solar cells to improve performance.	Methyl-ammonium lead iodide vs. quadruple-cation perovskites	Combination of steady-state and transient PL, general recombination model simulation, normalization of transient PL curves at 50 ns, sensitivity analysis of trap parameters.	Significant variability in trap parameters across studies, uncertainties in experimental methods.	Quadruple-cation perovskite shows better performance due to reduced electron capture coefficient, not lower trap density. Steady-state PL intensity is sensitive to trap density.
Yuan et al. [41]	Recombination dynamics in halide perovskites to optimize solar cell performance.	Halide perovskites	Steady-state and transient PL, time-correlated single-photon counting, gated CCD, dynamic range control via filters, SCAPS software for band diagrams, MATLAB simulations using coupled rate equations.	Experimental complexity, requiring multiple methods and software for accurate analysis.	Detailed analysis of recombination dynamics and quasi-Fermi-level splitting. PL simulations provide insights into recombination processes in perovskite films, aiding in solar cell optimization.
Zhang et al. [42]	Challenges in explaining PL behavior in MAPbI_3_ thin films, focusing on film thickness (L).	MAPbI_3_ perovskite thin films	Steady-state PL spectra, PL decay kinetics with bi-exponential fitting to distinguish surface and bulk recombination, correlation of film thickness with trap density and charge transport properties.	Difficulty in isolating surface vs. bulk contributions to PL due to overlapping processes.	Thicker films show longer PL lifetimes due to reduced trap density and improved charge transport. Red-shifts in PL spectra with increasing thickness indicate changes in grain size and electronic environments.

## Data Availability

Data are contained within the article.
